# Is Piric Acid a Reliable and Practical Test for Albuminuria?

**Published:** 1885-01

**Authors:** Frank Cary

**Affiliations:** 3027 Indiana avenue; Chicago


### ARTICLE III.

### IS PICRIC ACID A RELIABLE AND PRACTICAL TEST FOR ALBUMINURIA? *By* FRANK CARY, M. D., *Chicago.*

As this test seems still before the profession—judging from the articles that from time to time appear in the different magazines, as a rule, eulogizing it—I beg leave in this JOURNAL to express my convictions concerning its utility for the general practitioner. Last year, while experimenting with the test, I noticed an allusion, in this JOURNAL to the "New Test for Albumen," proposed by Dr. Johnson. This "new test" consists in floating on the surface of a clear urine—which, we are instructed, must be neutral or slightly acid, but which may, I think, be alkaline as well—a saturated aqueous solution of picric acid. This, having a specific gravity of about 1003, can, with care, be floated on the urine without the two liquids becoming mixed. Should there be albumen present, a pale opalescent line will appear at the junction of the two liquids, as in Heller's test. This picric acid test, that has caused no little discussion, is no "New Test." It is spoken of in Quain's Medical Dictionary as a confirmatory test, and has been in use at the Westminster Hospital for the past eight or nine years.* One of the principal advantages claimed is its superior delicacy over the heat and nitric acid tests.

Dr. George Oliver,† who places the nitric acid test below that of acidulated brine, and above all in point of delicacy, the picric acid, mercuric iodide, and his own test papers, states that "the mercuric salts are said to cause a quasi albuminous precipitate in the urine of patients taking alkaloid," but that he is not "convinced of the correctness of this assertion." Though he found by using the mercuric iodide test, albumen present in all the patients, he mentions as taking alkaloids, he says: "The same reaction followed the use of picric acid"—here seeming to assume that picric acid would not precipitate alkaloids in solution.

In twenty cases reported by him he states that he failed to detect albumen in sixteen with cold nitric acid (Heller's test), fourteen with heat and acid, while with picric acid, and potas-

---

* Lancet, May 19, 1883, p. 886.

† Lancet, January, 1883.

sic mercuric iodide he obtained a "distinct and generally a sharply defined ring of precipitated albumen in every case." My results with picric acid have been similar to the above-mentioned twenty cases. Holding a test tube at an angle of about 45° and allowing the solution to flow slowly down its side, one is able to float the acid on the surface of the urine with the least possible admixture, and in an examination of some sixty specimens from persons in apparent health—more than half being students attending Bellevue—I was able in every instance—great care being exercised to keep the two liquids separate—to detect a more or less distinctly marked opalescent line. From accumulated evidence, there can now be no doubt entertained as to the various alkaloids—quinine, aconite, pilocarpine, etc., causing a marked precipitate in the urine of patients taking these drugs in any quantities—and urates when in excess, as well as peptones, are also precipitated, producing the pale line. Some who favor the test suggest that if the precipitate be thought to come from urates, the microscope could be called in to settle the question, if from alkaloids, the addition of a little alcohol, nitric acid, or gentle application of heat. Any of these procedures require time, and in our manipulations we are likely to destroy the line of contact between the liquids.

Suppose, on examining a specimen, you detect a faint line just where the acid and urine meet, you are in doubt as to just what produces it, whether it is coagulated albumen, or precipitated alkaloid. You resort to heat, and with what result? The line disappeared just before the boiling point was reached, but you are in doubt as to whether it was "cleared up" or dissipated by the mixing of the two liquids. Can heat be applied to two liquids in a test tube and they not be more or less mixed? As the particles near the source of heat become heated they must of necessity rise, their places being taken by the cooler particles from below, and in this way will the line be broken up and lost. Dr. Robert Kirk, who has used this test for some time, states* that unless it be used as Heller's test, *i. e.*, keeping the two liquids separate, it is "valueless." Though I do not say the test has no value—believing it has—I am, nevertheless, convinced that it has so many drawbacks as an "every-day test," that, for the general practitioner, it is worse than valueless, being liable to give results which can hardly fail of producing errors in diagnosis.

3027 Indiana avenue.

---

### ARTICLE IV.

A SIMPLE MICROTOME. *By* C. C. WEBSTER, M. D.

Several years ago, Mr. O. B. Marsh, of Binghampton, N.Y., made, at my suggestion, the herein described instrument. I have found it very useful, particularly in cutting sections of malignant growths for diagnosis. The frequent necessity for such an instrument leads me to publish the description, with

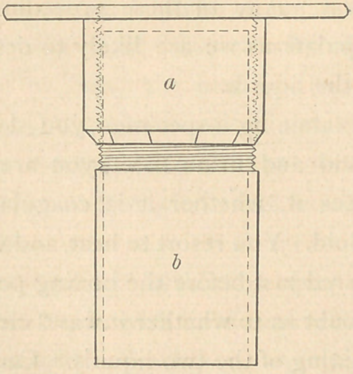

*a. Nut.*
*b. Hollow Screw.*

---

*London Lancet, April, 1883.

